# Elevated serum concentrations of activated hepatocyte growth factor activator in patients with multiple myeloma

**DOI:** 10.1111/j.1600-0609.2008.01130.x

**Published:** 2008-11

**Authors:** K F Wader, U M Fagerli, R U Holt, B Stordal, M Børset, A Sundan, A Waage

**Affiliations:** 1 Department of Cancer Research and Molecular Medicine, Faculty of Medicine, Norwegian University of Science and TechnologyTrondheim; 2 Department of Oncology, St Olavs HospitalTrondheim; 3 Department of Food and Medical Technology, Sør-Trøndelag University CollegeTrondheim; 4 Department of Immunology and Transfusion Medicine, St Olavs HospitalTrondheim; 5 Department of Hematology, St Olavs HospitalTrondheim, Norway

**Keywords:** multiple myeloma, hepatocyte growth factor, scatter factor, hepatocyte growth factor activator

## Abstract

**Objectives:**

Hepatocyte growth factor (HGF) is a potential key factor in multiple myeloma. Conversion of pro-HGF to its active form is a critical limiting step for its biological effects. We aimed to examine the levels of the most potent activator, the hepatocyte growth factor activator (HGFA), in serum and bone marrow plasma of patients with multiple myeloma.

**Methods:**

The activated form of HGFA was measured by an enzyme-linked immunosorbent assay in serum (*n*= 49) and bone marrow plasma (*n*= 16) from multiple myeloma patients, and in serum from healthy controls (*n*= 24).

**Results:**

The median concentrations of activated HGFA in myeloma and control sera were 39.7 (range 6.2–450.0) and 17.6 ng/mL (range 4.8–280.6), respectively. The difference was statistically significant (*P*= 0.037). The median concentration of activated HGFA in bone marrow plasma was 6.1 ng/mL (range 3.5–30.0).

**Conclusion:**

We here show for the first time that the activated form of HGFA is present at high levels in serum and bone marrow of myeloma patients, thus providing a necessary prerequisite for the activation of HGF.

Hepatocyte growth factor (HGF) stimulates survival, proliferation (1), adhesion (2) and migration (3) of malignant plasma cells and is a potential contributor to the bone disease of multiple myeloma (4). HGF is produced by myeloma cells and by stromal cells in the bone marrow microenvironment, and thereby acts in an autocrine or paracrine manner through its receptor c-Met (5–7). We and others have previously shown that serum HGF levels are elevated in myeloma patients compared with normal controls, and associated with poor prognosis (8, 9).

HGF is secreted as a single chain precursor which is proteolytically converted to its biologically active heterodimeric form. The most potent activator is the factor XII-related serine protease hepatocyte growth factor activator (HGFA) (10, 11). HGFA is mainly secreted by the liver, although extrahepatic expression has been reported in a number of normal and tumour tissues (12). It circulates in plasma as a single-chain 96-kDa pro-form, which is activated by thrombin in the presence of negatively charged molecules to its 34-kDa active two-chain heterodimeric form (13). The HGFA activity is regulated by the HGF activator inhibitors (HAI)-1 and -2, reviewed in (12).

Tjin *et al.* (14) showed that myeloma cells express HGFA and thereby proteolytically convert single chain HGF into its active form. We aimed to examine the levels of the activated form of HGFA in serum and bone marrow plasma from myeloma patients, and to correlate the serum levels with clinical stage, parameters of disease activity and survival. Secondly, we aimed to investigate a possible relationship between the concentrations of HGFA and HGF.

## Patients and methods

We examined serum samples drawn at diagnosis from 49 patients diagnosed with multiple myeloma in mid-Norway between 1996 and 2005. We also examined bone marrow plasma from the same patients when available (*n* = 16). Serum and bone marrow plasma samples were drawn before initiation of treatment and frozen at −80°C until they were analyzed. In six patients, we also examined serum drawn at time of first response, defined according to the EBMT/IBMTR/ABMTR criteria (15) and at first relapse, defined as the time point where treatment was re-introduced. Control samples were obtained from 24 healthy volunteers. Because of limited quantities of sample material, HGF was analyzed in only 20 of the 24 controls. Clinical information about the myeloma patients was obtained retrospectively from the patient records. Registered information was stage according to Durie Salmon and International Scoring System (ISS), type and concentrations of serum and urine M-component, plasma cell percentage in bone marrow aspirate, serum β_2_-microglobulin and overall survival. The study protocol was approved by the Regional Medical Ethics Committee and the study was performed according to the declaration of Helsinki.

The median age of the myeloma patients (33 men and 16 women) was 65 yr (range 30–87 yr), and of the controls (15 men and 9 women) was 68 yr (range 44–81 yr). The patients were representative of the general myeloma population with serum M-component of IgG type in 29 patients (59%), IgA in seven patients (14%), other Ig isotypes in three patients (6%), only light chain secretion in nine patients (18%) and non-secretory myeloma in one patient (2%). Twenty patients (41%) were in stage 1 according to ISS, 13 patients (26%) in stage 2 and 11 patients (22%) in stage 3; for five patients (10%), no information was available.

We used a commercially available enzyme-linked immunosorbent assay (ELISA) for the measurement of activated HGFA (IBL, Gunma, Japan) in serum and bone marrow plasma. The assay was performed according to the manufacturer’s instructions. All samples were run in duplicates. The standard curve was linear between 0.9 and 15 ng/mL, and samples were diluted to concentrations within this range. The intra-assay and interassay variation coefficients for this assay are 5.5% and 5.5% at 6.5 ng/mL according to the manufacturer. Variation coefficients for our measurements were <10%.

HGF was measured with an ELISA from R&D systems (Minneapolis, MN, USA). The assay was performed according to the manufacturer’s instructions. All samples were run in duplicates. The standard curve was linear between 0.5 and 8 ng/mL. Because of limited quantities of sample material, the measurements could not be repeated and therefore samples with HGF concentrations lower than 0.5 ng/mL and above 8 ng/mL were given the values 0.5 and 8 ng/mL. Variation coefficients for our measurements were <10%. Up to two freeze–thaw cycles of serum did not affect the measured levels of HGF or HGFA.

SPSS Statistical Software version 14.0 was used for statistic calculations (SPSS Inc, Chicago, IL, USA). Comparisons between groups were performed by the Mann–Whitney *U*-test. Correlations between two parameters were estimated by Spearman’s rank correlation analysis. Survival analysis was conducted by the Kaplan–Meier method, using median values as cut off. The level of statistical significance was set at *P*< 0.05. *P*-values were two-tailed.

## Results

**Figure 1:**
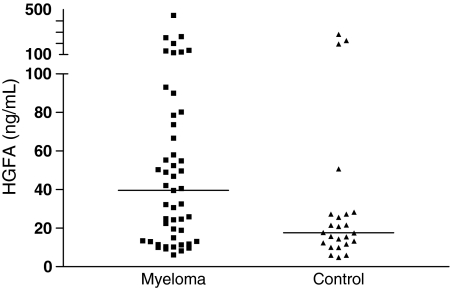
Serum concentrations of activated HGFA measured by ELISA in 49 multiple myeloma patients and 24 age- and gender-matched controls. Bars indicate median concentration.

Serum levels of activated HGFA in patients at the time of diagnosis and in controls are shown in Fig. 1. The median HGFA concentrations in myeloma and control sera were 39.7 (range 6.2–450.0) and 17.6 ng/mL (range 4.8–280.6), respectively. The difference was statistically significant (*P*= 0.037). The median level of activated HGFA in bone marrow plasma of myeloma patients was 6.1 ng/mL (range 3.5–30.0) (data not shown). Thus, HGFA levels were lower in bone marrow plasma than in serum. However, serum and plasma HGFA levels cannot be directly compared, as measurement of levels in serum will be 2–3 times higher than in plasma in this assay according to the manufacturer and own validation experiments (data not shown).

There was no correlation between the serum levels of HGFA and disease stage according to ISS or Durie Salmon, concentration of serum M-component, serum β_2_-microglobulin, percentage of plasma cells in the bone marrow or overall survival (data not shown). The HGFA levels did not covariate with disease activity in serial measurements of serum drawn at diagnosis, remission and relapse in six myeloma patients (data not shown).

**Figure 2:**
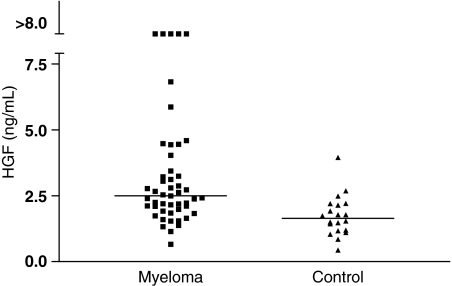
Serum concentrations of HGF measured by ELISA in 49 myeloma patients and 20 age- and gender-matched controls. Bars indicate median concentration.

The median HGF concentrations in myeloma and control sera were 2.5 (range 0.7–8.0) and 1.6 ng/mL (range 0.5–4.0), respectively (Fig. 2). The difference was statistically significant (*P*< 0.001). The median HGF concentration in bone marrow plasma was 8.0 ng/mL. There was no correlation between the levels of HGFA and HGF in serum (*r*_s_ = 0.14, *P*= 0.26) or bone marrow plasma (*r*_s_ = 0.31, *P*= 0.38).

## Discussion

HGF has a number of myeloma-relevant activities; however, it has to be converted to its heterodimeric form to be biologically active. Urokinase-type plasminogen activator (uPA), tissue plasminogen activator, factor XIIa and matriptase have all been shown to activate single chain HGF at low rates (11, 16–18). The most potent activator is, however, the factor XII-related serine protease HGFA, with an HGF-converting potency of more than 1000 times that of uPA (11). Tjin *et al.* (14) showed that myeloma cells express HGFA, thereby activating HGF. We here demonstrate for the first time that HGFA exists in its activated form in serum from myeloma patients, and that serum concentrations are higher than in healthy controls. We also found detectable activated HGFA in 16 of 16 samples of bone marrow plasma from myeloma patients.

The role of HGFA in regulating HGF activity in injured tissue is well established (12). Recent data support an important function of HGFA also in solid tumours such as colorectal cancer (19) and glioblastoma (20). Among lymphomas, the HGF receptor is predominantly expressed in diffuse large B-cell lymphoma (DLBCL), and interestingly, DLBCL cells also express HGFA, possibly activating HGF produced by macrophages in the tumour microenvironment (21).

The activity of HGFA is tightly regulated. Secreted as an inactive single chain pro-form, cleavage by thrombin is essential for its function. In a recent publication, the kallikrein-related peptidases 4 and 5 were shown to have HGFA-activating properties similar to thrombin (22). The activity of HGFA is also controlled by the Kunitz type serine protease inhibitors HAI-1 and HAI-2 (12).

It is possible that the myeloma cells directly contribute to the elevated HGFA levels in serum of myeloma patients. However, we found no correlation between the serum HGFA concentration and disease stage or traditional markers of tumour burden. As we have measured only the activated form of HGFA, the elevated levels in myeloma patients might also mirror a higher degree of activation of pro-HGFA in patients compared with controls. The complex mechanisms regulating activation of HGF in multiple myeloma, including a potential role for the HGFA inhibitors HAI-1 and HAI-2, should be addressed in further studies.

We found no correlation between serum levels of HGFA and HGF. This is partly in disagreement with Nagakawa *et al.* (23), who found a positive correlation between serum levels of HGF and HGFA in patients with untreated and advanced stage prostate cancer. However, the fact that we have measured total HGF, which is both single chain HGF and the active heterodimer, may obscure a positive correlation between HGFA and active HGF.

In conclusion, activated HGFA is present at high levels in serum and bone marrow of myeloma patients. Although this study has obvious limitations because of the relatively small number of study subjects, it clearly demonstrates the presence of a necessary prerequisite for activation of the HGF system in multiple myeloma. It also points to the activation step of HGF as a possible target for therapeutic intervention.
